# Total-body positron emission tomography imaging to accelerate radiotracer discovery pipelines

**DOI:** 10.1016/j.pharmr.2025.100066

**Published:** 2025-05-15

**Authors:** Andrew Sutherland, Marc R. Dweck, David E. Newby, Adriana A.S. Tavares

**Affiliations:** 1 School of Chemistry, University of Glasgow, Glasgow, United Kingdom; 2 British Heart Foundation-University of Edinburgh Centre for Cardiovascular Science, University of Edinburgh, Edinburgh, United Kingdom; 3 Edinburgh Imaging, University of Edinburgh, Edinburgh, United Kingdom

## Abstract

The development of the first total-body positron emission tomography (PET) clinical scanner is a transformational moment in nuclear medicine, reigniting the field by tackling 2 long-standing and critical barriers to the widespread clinical use of PET: radiation dose and patient throughput. Total-body PET also provides several other unique research and clinical opportunities, including potential to streamline radiotracer discovery and development pipelines. PET does not exist without radiotracers. However, despite decades of radiotracer development programs, the number of successful PET radiotracers adopted and approved for human use is extremely low. In neurology, an important area for nuclear medicine, only approximately 4% of all novel radiotracers that survive the radiotracer translational “valley of death” are adopted clinically. The potential for total-body PET technology to reverse these low numbers of radiotracer development and adoption is high. This will require the PET community to come together with the regulators to chart new frameworks for radiotracer development and translational pipelines. This article will discuss which stages of the radiotracer discovery pipeline can benefit most from the recent development of total-body PET technology. It will review the latest key developments in radiochemistry modernization and describe how these could ameliorate regulatory hurdles and deliver the groundbreaking potential of total-body PET. Finally, this article will highlight emerging radiotracer discovery opportunities that could be rapidly facilitated by total-body PET.

**Significance Statement:**

In addition to creating new opportunities for clinical research and patient care, total-body positron emission tomography technology can also embolden radiochemistry modernization in the clinic and break long-standing translational barriers encountered during radiotracer discovery pipelines.

## Graphical abstract

**Figure:**
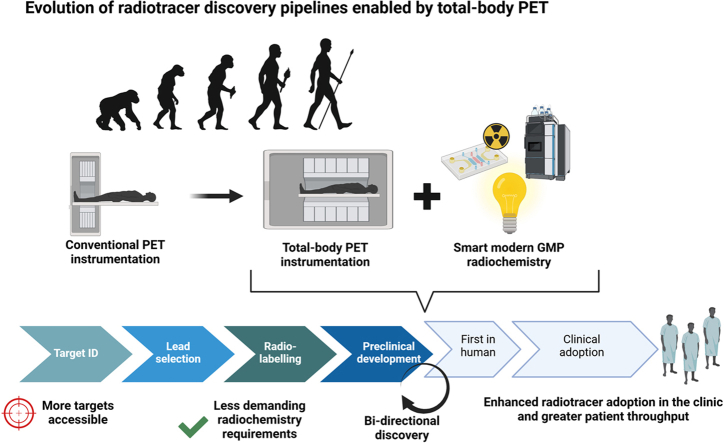

## I Introduction

The successful construction of the first total-body positron emission tomography (PET) scanner in 2018^1^ represents a transformative step in the fields of nuclear medicine and whole-person research. The concept of total-body PET imaging was introduced in 1990 as a rotating system around the research subject,^2^ but did not materialize as a commercially available and regulatory compliant scanner until 2018. Total-body PET imaging is defined as the ability to scan the whole human body simultaneously at any given moment. This is distinct from whole-body PET imaging, which refers to the ability to image the whole human body by moving the scanner bed through a ring of PET detectors and has been available as an imaging tool for over 30 years. Conventional PET systems have an axial field of view (FOV) of approximately 20–30 cm, whereas total-body PET systems have an axial FOV of 200 cm. Large or long-axial FOV PET systems (typically 100–150 cm) have also been developed since 2018. The increased PET ring coverage of these large axial FOV systems versus conventional PET systems has vastly improved scanner imaging performance, in terms of sensitivity and signal-to-noise ratio.^1^^,^^3^^,^^4^ Theoretically, the sensitivity gains could be as high as 40 times compared to conventional PET scanners with a signal-to-noise ratio improvement of ∼6-fold.^1^

Imaging sites housing the first large axial FOV PET systems have now started reporting the feasibility of low and ultralow-dose imaging studies while retaining quantitative accuracy and image quality of comparable diagnostic quality to conventional PET systems.^5^^,^^6^ Through innovative “computed tomography-free” attenuation map techniques,^7^, ^8^, ^9^, ^10^, ^11^ quantitatively reliable PET imaging can be achieved with radiation doses under 0.5 mSv. This would take PET from one of the most dose-intensive modalities to being of similar magnitude as planar radiographs.^9^^,^^12^ The implications of this major technological development on radiotracer discovery programs are substantial because regulatory frameworks for radiotracer translation to humans require risk-benefit analysis, which would be significantly abridged when using large axial FOV PET systems for first-in-human investigations. Furthermore, it is known that discussions about radiation dose can be emotive,^13^ in particular in some medical fields such as pediatrics and obstetric imaging. Reduction of radiotracer injected doses coupled with “computed tomography-free” attenuation protocols can make PET a more viable technique in these contexts,^14^, ^15^, ^16^ galvanizing new radiotracer discovery programmers focused on clinical populations that have been under-targeted thus far. Importantly, lower radiation doses would allow the use of PET imaging as a sensitive screening tool,^17^ facilitating radiotracer development programs focused on characterizing human physiology throughout the lifespan. This is an area of great need in view of the rapidly aging populations and the limited classification of the impact of natural aging on human physiological systems.^18^ Most radiotracer development programs thus far have focused on pathological applications and targets. However, with the advent of large axial FOV PET scanners, targeting fundamental biology and physiology for radiotracer discovery programs is likely to increase and symbiotically enhance our understanding of pathophysiology versus normality datasets. For example, recent radiotracer development programs for metallomics^19^ and vitamin research^20^, ^21^, ^22^ illustrate the value of harnessing new opportunities in the total-body PET era.

The PET radiotracer discovery and development community has a critical challenge ahead, to seize this opportunity and innovate further, but also to reshape regulatory frameworks for radiotracer translation to humans. This requires a change in community views to enable widespread clinical adoption, in light of the new and exciting characteristics of total-body PET technology described earlier. After all, PET does not exist without PET radiotracers. Despite the obviousness of this statement, the success rates of developing and translating new radiotracers have been low. It is known that the preclinical stages of radiotracer discovery are some of the most challenging with only ∼30% success rate.^23^ This is lower than the first-in-human success rate that averages at ∼75%^23^^,^^24^ but higher than clinical adoption following first-in-human studies. For example, according to the National Institute of Mental Health, 138 brain PET radiotracers have been used in human studies. Of these, only 6 have successfully secured Food and Drug Administration approval, which averages at a ∼4% success rate in terms of clinical adoption and market authorization. This article will review the classical milestones in the radiotracer development pipeline and highlight which stages of the pipeline can benefit most from the recent development of total-body PET technology. It will also review the latest developments in radiochemistry modernization and describe how these could ameliorate regulatory hurdles and aid the delivery of the groundbreaking potential of total-body PET. Finally, this article will highlight emerging radiotracer discovery opportunities that could be rapidly catalyzed by total-body PET. Because the development of total-body PET technology is still very recent and there is a relatively limited amount of literature on this topic, especially in the context of total-body PET impact in radiotracer development, this review paper will provide insights on how total-body PET could resolve the many challenges in radiotracer development in the coming years. This is because the process of developing and translating new radiotracers is a lengthy one, and thus, robust quantification of the impact of this technology on the radiotracer development pipeline will require decades of total-body PET use.

### A Conventional radiotracer discovery pipeline

**Fig. 1:**
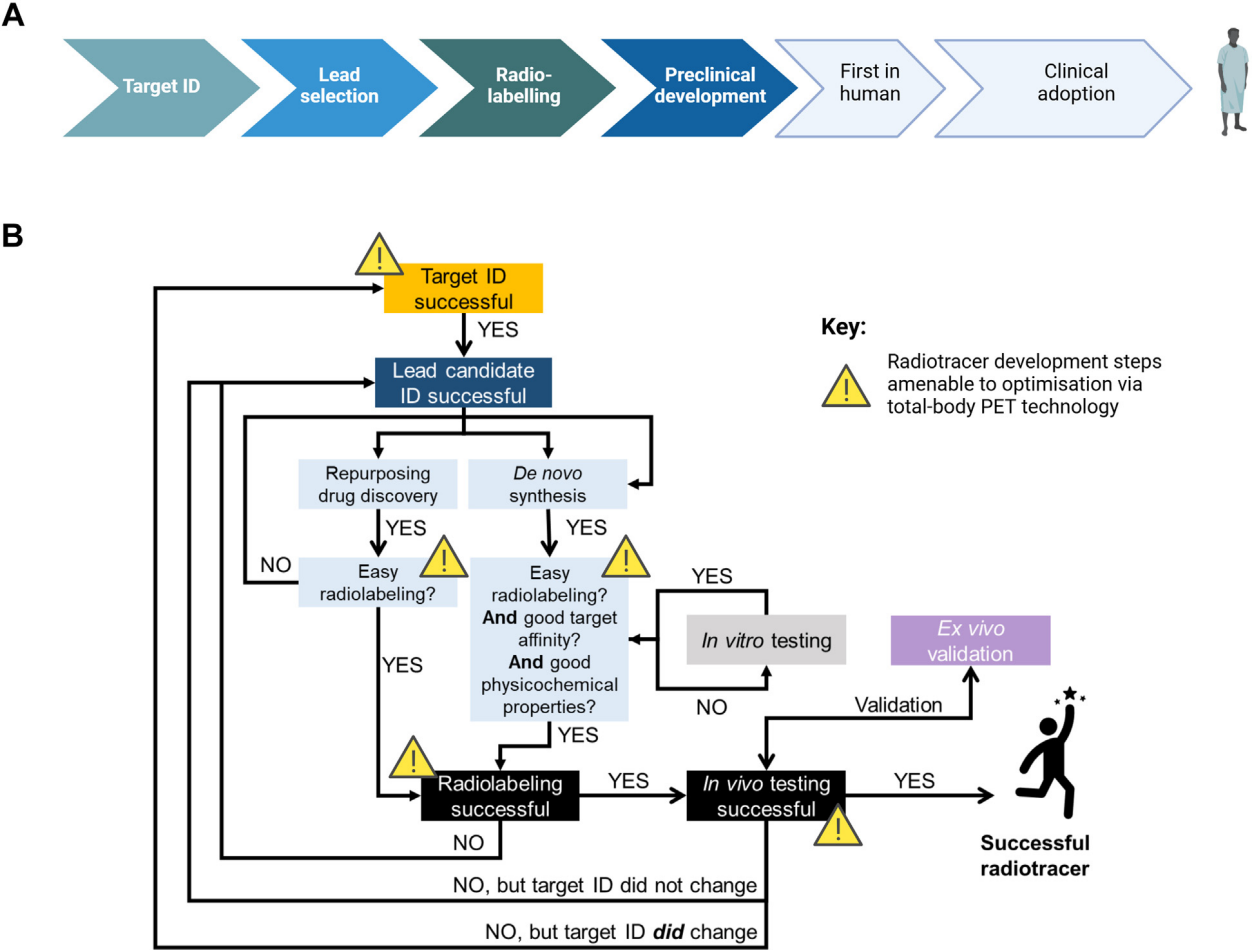
Conventional radiotracer development pipeline. Main steps associated with radiotracer development pipelines (A) and typical flowchart illustrating suggested approach to radiotracer discovery and development (B). Radiotracer development steps amenable to optimization via total-body PET imaging are highlighted in panel (B). Central to this optimization is the role that total-body PET technology can play in modernizing radiochemistry activities in the clinical environment. Panel (B) modified with permission from Shaw et al.^23^

Others and we have previously described the classical radiotracer discovery pipeline in detail (Fig. 1A),^23^^,^^25^, ^26^, ^27^, ^28^ and this is outside the main scope of the current review paper. However, to better contextualize current knowledge of typical radiotracer discovery pipelines and better support understanding of subsequent sections in this review article, we here provide a brief overview of the key steps associated with developing new PET radiotracers:

• **Target identification.** The journey of radiotracer discovery and development starts with scientific, clinical, and marketing scoping exercises. Typically, this means investigating which target(s) play an important role in a given disease and can be imaged using PET (considering, eg, the test−retest variability of the imaging technique). Targets that play important roles in multiple disorders can broaden the range of applications of the novel radiotracer candidate, thereby making them more attractive targets. In the classical radiotracer discovery pipeline, target density is an important factor when establishing acceptance criteria for radiotracer binding properties, including affinity to the target. In addition to clinical and scientific scoping, it is also important to consider whether the proposed radiotracer program would attract investment and lead to commercialization, thereby enabling market authorization for efficient clinical routine use.
• **Lead compound identification.** The design and selection of the lead radiotracer candidate can start by targeting existent therapeutic drugs or initiating a de novo radiotracer program. At this stage, it is important to consider targeting compounds that allow for ease of radiolabeling. If a de novo approach is favored then a synthetic route is designed for late-stage diversification, resulting in a range of final compounds that can be used to develop a structure activity relationship with the biological target. Libraries of compounds are designed, synthesized, and screened for key properties, such as affinity to a given target and lipophilicity, with the view of rapidly identifying the lead candidate for progression into in vivo characterization.
• **Preclinical development.** At this stage of the radiotracer discovery and development pipeline, controversies can arise when discussing which animal species to use and often a case-by-case scientific analysis is necessary, given divergent target expressions in humans versus preclinical species such as mice, rats, or nonhuman primates. Furthermore, some animal models such as the mouse or rat are more amenable to genetic manipulation versus other preclinical species, which may be advantageous in some pathologies with target expression only detected as a consequence of genetic mutations. In recent years, a growing consensual view exists that moving straight into in vivo preclinical imaging and skipping in vitro/ex vivo testing is the most efficient way to proceed. However, this is currently being challenged as nonanimal methodologies^29^ gain momentum. Nonanimal methodologies, which typically are in vitro/ex vivo testing approaches, are currently being proposed as alternatives to animal experimentation and precursors to first-in-human studies. The preclinical stage of radiotracer development is very iterative and failure prone, often leading back to earlier stages of radiotracer discovery pipelines (eg, target identification or lead compound selection).
• **Clinical development.** Once a radiotracer successfully moves through the preclinical stages of development, the first challenge to overcome is species differences in metabolism and binding kinetics to target. This is often referred to as the translational “valley of death” and intuitively is the least predictable barrier to success.^30^ Other hurdles need to be considered during the clinical development stages, including regulatory requirements.^23^ In this regard, it is concerning to observe that, for example, the time between successful radiolabeling and human studies with new radiotracers increased from 1–2 to 5–10 years over the past 40 years of neuroreceptor imaging.^31^ Most of this radiotracer development timeline expansion was due to an increase in time to translate new radiotracers to humans as well as progressing through the clinical development stages until clinical adoption. Further issues include the ever-growing and often confusing legislation for clinical development.^32^^,^^33^ By contrast, the time taken to pass through preclinical development has been reduced due to the introduction of preclinical PET imaging early in the pipeline. Regulatory approval is also extremely diverse depending on geography, as reviewed by Schwarz et al^34^ in 2019. This can have a significant impact on timelines and costs of translating new radiotracers to humans, as well as the development of these into clinically viable products for routine use.

Looking at the conventional radiotracer discovery pipeline, it is clear that each step has challenges and barriers to success. The lowest success rate during radiotracer development pipelines is at the stage of clinical adoption (4%), followed by preclinical development (30%) and preclinical to clinical first-in-human translation (75%). Total-body PET could significantly aid with increasing clinical translation and, importantly, adoption of new PET radiotracers. In the next section, we discuss different ways in which total-body PET could reshape the traditional radiotracer discovery and development pipelines.

### B Redefining radiotracer discovery pipelines with total-body positron emission tomography

The first stage in the radiotracer discovery pipelines is target identification. Often, low expression targets are either deprioritized or fail to succeed due to the signal-to-noise ratio limits of conventional PET scanners.^23^^,^^35^ The use of total-body PET systems, with several fold improved signal-to-noise ratios,^1^ could widen the range of biologically meaningful targets for radiotracer development (Fig. 1B). Concomitantly with the identification of biologically meaningful targets, the choice of chemical moieties for radiotracer development is key, as that determines the likelihood of success of radiochemistry methods. This is because molar activity and radiochemical yield are key outcome metrics when designing radiosynthetic routes for new radiotracers. Given the higher sensitivity of total-body PET systems compared with conventional PET scanners, radiotracer production methods with low radiochemical yield gain feasibility as suitable for human use. It would also be possible to extend the radiosynthesis reaction times closer to nonradioactive organic chemistry methods, thereby accelerating translation of chemistry methodology (Fig. 1B). Importantly, the use of less expensive precursor material for good manufacturing practice (GMP) procedures becomes accessible. Even if the radiochemical yields are low, these would be efficient enough for total-body PET imaging.^35^ The use of total-body PET technology could also assist with the molar activity requirements, because high molar activity could be compromised for faster human translation, while still operating within the microdosing principle, or could take advantage of high molar activity radiotracer to explore low-density targets with high sensitivity^35^ (Fig. 1B). Furthermore, the high sensitivity of total-body PET systems means that the cost analysis of PET imaging becomes more attractive than conventional models,^36^ because it would be possible to lower the radiotracer injection dose and/or image more quickly, allowing more patients to be scanned per day.

**Fig. 2:**
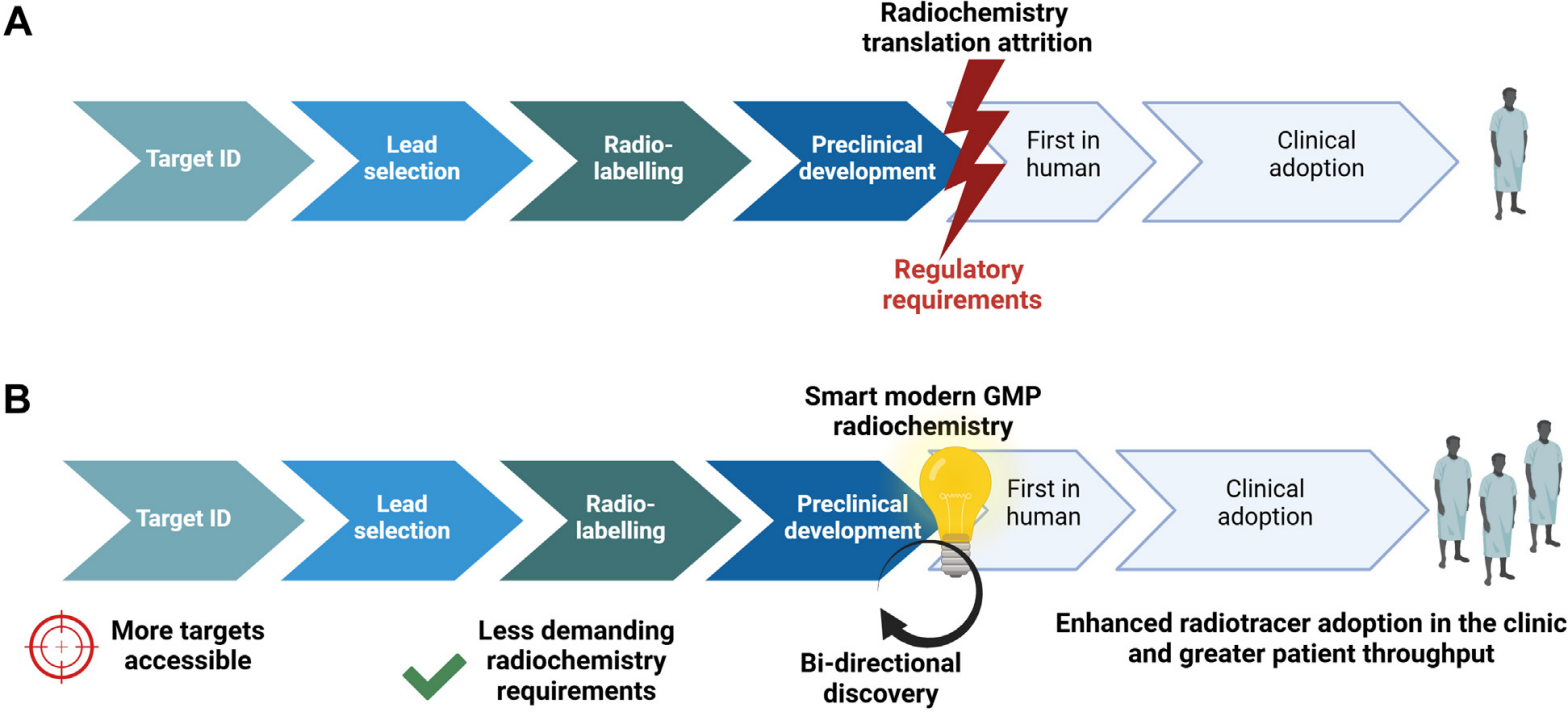
Redefining radiotracer discovery pipelines with total-body PET. Comparison of conventional (A) and total-body PET redefined radiotracer development pipelines (B). The development of total-body PET technology allows for interrogation of more biologically meaningful targets given the total-body PET scanners’ improved sensitivity. Furthermore, total-body PET can facilitate radiolabeling approaches and, through smart and modern GMP radiochemistry methodologies, can streamline first-in-human studies with new radiotracers. Ultimately, this would lead to enhanced radiotracer adoption in the clinic and greater patient throughput. A greater bidirectional translation activity will be possible, galvanizing discovery science.

An argument could be made for the use of total-body PET imaging to circumvent the use of animal models and earlier go/no-go decisions before launching expensive clinical trials. This is based on the possibility of obtaining pharmacokinetics and human dosimetry using radiation doses that are below the annual natural background levels^1^ However, these methods are yet to be established, especially as more evidence emerges of to-date unknown human biodistribution patterns for old PET radiotracers such as [^18^F]fluorodeoxyglucose,^37^ which require in-subject laboratory tissue validation studies that are overall challenging or not feasible in living humans. Furthermore, despite the tremendous potential of total-body PET to push the frontiers of microdosing principles to nanodosing principles, a degree of simplified yet important preclinical studies would be prudent. Even though safety should not be the stumbling block in nanodosing and microdosing studies, the greater sensitivity of total-body PET would unveil new biology that merits laboratory validation to better use new PET radiotracers. It is possible the translational phase in radiotracer development pipelines would be more bidirectional with total-body PET enabling greater advances in discovery science (Fig. 2). Preclinical studies in radiotracer development could also be of importance for a range of particulates found in consumer products and the environment because these cannot be easily studied in humans given the multifactorial nature of these targets and the difficulty to control for confounders in humans. Furthermore, many of these particulates are everlasting materials, which present some ethical challenges.

An important quantitative bias of increasing importance in total-body PET radiotracer development experiments is the adhesion of radiotracer to the injection lines and arterial blood sampling lines in first-in-human studies.^35^ Radiotracer adhesion to lines is a well-known phenomenon to consider in PET imaging; however, the longer axial FOVs of total-body PET scanners amplify this bias. Although adhesion to injection lines can be fairly easily addressed by measuring residual activity in lines postinjection, the adhesion to arterial lines is a greater challenge. In part, arterial line adhesion could be resolved by using image-derived radiotracer concentration time−activity curves in arterial pools, but a challenge remains for radiometabolite analysis, which is required during radiotracer development programs. Multiple strategies could be considered to alleviate this challenge, including using image-derived radiometabolite models,^38^, ^39^, ^40^, ^41^, ^42^ but these techniques are still in their infancy in the field of PET imaging. A more practical approach to minimize arterial blood collection line bias could be to test and identify the line materials that are least susceptible to radiotracer adhesion. For example, polyethylene lines are known to have greater adhesion than polytetrafluoroethylene lines.^35^

Given the significant costs associated with achieving regulatory compliance during radiotracer translational pipelines, there are relatively few medical centers that can implement new radiotracers.^43^ A few strategies could be considered to alleviate this bottleneck in radiotracer development, including lobbying regulatory bodies to reassess current requirements for regulatory submission packages for new radiotracers in view of total-body PET’s improved sensitivity, enabling lower mass dose and radiation dose studies. These regulatory packages are a major burden in the process of radiotracer translation and are widely variable in different countries.^34^^,^^44^^,^^45^ Standardization and harmonization of these requirements would enable better global health care programs using PET imaging. Another important strategy would be to have total-body PET centers partnering as scientific consortia to conduct in-depth comparative radiotracer studies when multiple compounds are available for the same target, in order to benchmark the best ligand for subsequent clinical adoption.

### C Total-body positron emission tomography to overcome the radiotracer translation “valley of death”

During radiotracer translation from preclinical to clinical use, several challenges need to be overcome. First, radiochemistry techniques often need to be scaled-up and adapted to the GMP environment. Second, whole-body dosimetry analysis is typically required for subsequent clinical use, adoption and marketing. Third, regulatory approval packages require GMP test batches as the evidence of radiotracer quality for human use and dosimetry estimates together with toxicology packages from preclinical species. Total-body PET imaging offers an opportunity to overcome or to minimize these translational bottlenecks, especially for the rapidly emerging field of theranostics.^27^^,^^46^ As previously described, total-body PET imaging opens a wide range of possibilities with regard to radiotracer quality metrics such as radiochemical yield and molar activity. In addition to flexing aspects of classic radiotracer quality targets, total-body PET imaging can play a pivotal role in realizing the potential of new and emerging technologies for “low-cost” radiochemistry. Given that radiotracers could be injected in low doses into humans and still obtain quantitatively accurate PET data using total-body PET scanners, previously dismissed technologies such as microdroplet radiochemistry and microfluidics synthesis would be re-energized. This could resolve the first translational “valley of death” challenge, by removing the need to consider expensive radiochemistry scale-up for production of large doses of radiotracer for human use.

The traditional radiotracer discovery pipeline argument has been that whole-body PET imaging of humans with short-lived radioisotopes requires production of radiotracer batches with high doses via automated radiosynthesizers. However, this approach is very wasteful when only a small batch of radiotracer is needed for first-in-human radiotracer translational purposes. Droplet microreactor systems have been previously developed with comparable yields to conventional synthesizer technology, while using 10–100 times less reagents, requiring less synthesis time and occupying a fraction of the shielded cabinet footprint.^47^ The combination of these advantages over conventional automated synthesizers also makes these microreactor approaches extremely cost-effective for the production of small on-demand PET radiotracer batches typically required for first-in-human work. Furthermore, their production costs are sufficiently low to enable single-use, streamlining GMP requirements. A critical laboratory-on-a-chip review recently published by the Royal Society of Chemistry further highlights the benefits of this technology,^48^ which, now that total-body PET scanners are a clinical reality, could radically combat one of the key “valley of death” translational bottlenecks.

Traditionally, first-in-human studies with novel radiotracers require dosimetry estimates, usually from preclinical species, and clinical use is often held back until human dosimetry data can be acquired.^49^^,^^50^ In light of the total-body PET potential to obtain meaningful data with doses close to planar radiology and annual background levels,^9^^,^^12^ it would be important to reflect on the value of current dosimetry study requirements during radiotracer development pipelines. At a minimum, these should be reassessed and adapted to total-body PET realities by the regulatory landscape teams. A collaborative approach is needed among developers, scientists, regulators, manufacturers, and industry to navigate regulatory and licensing intricacies in the new total-body PET era. There is also a need to educate experts and nonexperts on radioactivity and put forward evidence-based action to change the negative image and the public’s perception of this topic, which is also often a barrier to PET radiotracer development and clinical use.^30^ This is particularly critical in the rapidly growing field of theranostics, where normal organ dose limits, individualized dosimetry, and radiobiological models are acutely needed but often hard to develop because of the onerous safety and efficacy requirements for first-in-human studies using conventional PET.^51^ The total-body PET low-dose paradigm shift could also aid in translating theranostic agents and more rapidly develop better models of dosimetry beyond classic radiotracer diagnostic compounds.

Toxicology packages, albeit mostly simplified for PET microdosing studies, remain very expensive. Because less mass could be injected into humans when using total-body PET versus conventional PET technology, it is possible to envision a new regulatory framework whereby further simplifications to the toxicology requirements could be deemed appropriate. This would consequently resolve another costly bottleneck in the “valley of death” translational space. For example, a concept of preclinical mass dose assessments using PET (eg, Knyzeliene et al^52^ and Tavares et al^53^) could be deemed appropriate in lieu of acute single-dose toxicology studies. The rationale in this scenario is that if the radiotracer is formulated in well-known nontoxic solutions and is shown to elicit no effects at a molecular target level via PET imaging, then the risk of inducing measurable toxic effects by classic tissue pathological analysis (current practice in toxicology studies) is negligible.

In addition to low radiation exposure and high patient throughput, total-body PET can be instrumental at supporting the development of new kinetic models for systems analyses and artificial intelligence-based multiparametric approaches. These new models and approaches can enable better description of new radiotracer pharmacokinetics and pharmacodynamics, thereby ensuring that successful radiotracers are not deprioritized due to complex kinetics. For example, the recently developed 18 kDa translocator protein-targeted radiotracer [^18^F]LW223 has divergent kinetics in the brain and heart,^54^ which required the development and validation of new models and multiparametric analysis to capture accurate translocator protein expression in the infarcted myocardium. Without these new models and approaches, successful radiotracers might be incorrectly deemed useless and deprioritized for further clinical use.

### D Total-body positron emission tomography empowered low-dose, low–cost, and radiochemistry modernization for radiotracer development pipelines

As alluded to in the previous section, the development of total-body PET scanners, along with the modernization of radiochemistry techniques in recent times, could act as a transformational shift in the field of PET, in particular radiotracer development projects. Without total-body PET’s high sensitivity and low-dose capabilities, most radiochemistry innovation in recent years would only be limited to preclinical settings. This is no longer the case. Droplet radiochemistry technology has exponentially grown since 2004 and has now overtaken batch and continuous production method publications.^48^ These low-cost techniques can now be produced by 3-dimensional printing, sterilized for single use and integrated into GMP radiochemistry laboratories. There are now several commercially available platforms for microfluidics GMP radiochemistry, and some compatible with quality control (QC) pipelines. In the context of radiotracer QC techniques in the GMP environment, traditional methods are costly and require a range of tests across multiple analytical instruments, including radio-high-performance liquid chromatography, radio-thin-layer chromatography, gas chromatography, and dose calibrator.^55^^,^^56^ Recently, Trace-Ability, Inc developed Tracer-QC, which is a single platform capable of conducting all necessary validation data from measurements produced by a plate reader. This platform can be fully shielded without a hot cell and receives the radiotracer from a shielded container,^56^ minimizing operators’ radiation exposure. Furthermore, it requires minimal setup as it relies on prepackaged kits and uses automated steps, reducing training requirements for operators and improving task reproducibility. This platform, which includes an integrated high-performance liquid chromatography, has already been used to validate radiotracers synthesized by microfluidics.^55^

Microfluidic synthesis modules coupled with an automatic QC platform could rapidly accelerate radiotracer translation and clinical use when a total-body PET scanner is available. These platforms could also be critical at delivering decentralized radiotracer production, thereby facilitating the adoption of new PET radiotracers in the clinical setting, because they require less skilled teams to run established and validated PET radiochemistry methodologies. The vision would be that a technician could simply load the raw radioactive material and relevant precursors and solvents, start the run, and receive a fully qualified human-ready dose of radiotracer. A detailed review describing the progress made on QC regulations and commercial equipment has been published.^56^ Delivering this vision would then enable faster and more efficient radiotracer translation programs. Still, adoption to these microfluidic approaches might take time. In the immediate future, rapid galvanization of the total-body PET potential in radiotracer discovery could be accomplished by adhering to fully automated standalone QC units that can be coupled with existing traditional PET radiosynthesizer infrastructure. In this regard, QC1 GmbH developed an ultracompact automated version of all necessary traditional equipment. This platform was acquired by Trasis in 2018 and was made commercially available in 2023. It relies on the use of kits that are loaded into the machine and generate QC results in around 30 minutes following the start of each run.^48^

Rapid and more efficient radiochemistry, especially with use of low-cost droplet technology, is now a reality in the clinical GMP setting of radiotracer development projects and is emboldened by total-body PET technology.

### E Role of total-body positron emission tomography in ameliorating regulatory hurdles and market authorization at the final stages of radiotracer discovery pipelines

A sense of anticipation exists in the community because total-body PET and radiochemistry advances collectively could represent a transformative shift in the landscape of nuclear medicine, especially in the context of radiotracer development. For example, the European Union (EU) has recently announced that nuclear medicine is a top priority in cancer research.^57^ This likely stems from evidence showing that the EU has decreased competitiveness compared with more flexible regulatory frameworks such as the United States, where more clinical trials have been approved in comparison with the “highly regulated” EU.^33^ In part, this relates with the pathways to obtain regulatory approval for first-in-human PET studies, which are less straightforward in the EU compared with the United States and Canada.^34^

A move toward harmonization of regulatory frameworks for market authorization is already underway for pharmaceutical drugs in the United Kingdom, where the Medicines and Healthcare products Regulatory Agency has launched an initiative in 2024 to facilitate safe access to new medicines with 7 international partners.^58^ If similar efforts could be developed in the context of radiotracer market authorization, inequalities in regulatory systems could be tackled more efficiently.

In the current climate of regulatory body harmonization and growing interest in nuclear medicine, the use of total-body PET technology could be a key enabler to streamline or remove bottlenecks at the final stages of radiotracer development and market authorization. This could be done by total-body PET consortia initiatives to conduct in-depth comparative radiotracer studies to declutter the “graveyard” of successful PET radiotracers and prioritize leads for clinical adoption with market authorization, as well as support for novel radiotracer development for a given target by head-to-head comparisons using a low- or ultralow-dose total-body PET paradigm. This is in opposition to the current practice of different centers using distinct subdeveloped radiotracers for clinical studies. Such an approach results in very protracted adoption of new and valuable PET radiotracers, leading to the disinvestment in radiotracer development programs by clinicians and regulatory bodies. This dampens support toward reimbursement campaigns for new radiotracers. Because total-body PET enables low-dose imaging, close to background levels, comparative radiotracer studies in humans become feasible as a way to prioritize the development of radiotracers for clinical adoption and reimbursement.

In the context of reimbursement, the cost of PET imaging has always been a bottleneck. Previous studies have shown that a cyclotron serving a single scanner is not financially viable^36^; therefore, strategies for decentralized radiochemistry (eg, droplet technology described earlier) should be considered in the new total-body PET era. Assuming modest levels of production of 12 radiotracer doses per day, the cost of radiotracer production was estimated to be $700/dose in 2001.^36^ Currently, in the United Kingdom, a dose of [^18^F]fluorodeoxyglucose from a central production facility ranges between approximately £250 and £500 (ie, $300–$600). By making use of total-body PET imaging properties, it would be possible to reduce the injected dose potentially by a factor of 10. Smaller batches of PET radiotracers would be required for clinical use, which would make radiotracer production more cost-effective. This corresponds to substantial savings in radiotracer costs enabled by total-body PET. Scaling this across the health care service would ameliorate new radiotracer adoption and augment market authorization appetite by the industry.

Obviously, a counter-argument exists for the financial impact of the higher cost of the total-body PET scanners versus conventional systems. Currently, the difference is approximately a factor of 7, depending on the large FOV PET scanner manufacturer. In 2001, the average cost of PET scans (technical scan and professional charges) ranged from approximately $900 to $1400, and the critical factor for profitability was throughput.^36^ Clinical centers with access to total-body PET scanners have reported an increase in patient throughput of at least a factor of 2 to 3 per day using available infrastructure originally designed for conventional PET scanner imaging. Although the savings from high-throughput scanning do not fully offset the scanner cost difference on their own, combined with radiochemistry savings, especially when using modern radiochemistry and automation, could majorly change the conventional PET costing models. This could resolve the bottleneck of clinical adoption of new radiotracers, which is mostly driven by high costs, long regulatory approval times, and expensive “niche” technical market.

### F Radiotracer discovery opportunities facilitated by total-body positron emission tomography

The low-dose nature of total-body PET is also crucial for opening new opportunities in the field of radiotracer discovery and development. For example, biologically meaningful targets in the pediatric population become viable and natural aging assessments gain significant momentum.^59^ Furthermore, imaging infection in situ, which is one of the greatest challenges in today’s world, gains special relevance in the context of total-body PET. It is known that patient treatment outcomes worsen when pathogens become resistant to commonly prescribed agents.^60^ In 2019, bacterial pathogens caused 7.7 million deaths globally, of which 1.27 million were directly linked to drug resistance.^61^ Despite multiple diagnostic techniques being deployed at the moment,^62^ infection-specific in situ imaging remains an unmet clinical need.^63^ Although conventional PET technology can already play a key role in the development of new PET radiotracers for imaging of infection,^24^^,^^64^^,^^65^ it is limited in its potential use in the context of intensive care medicine, a field where infection is particularly life-threatening.^66^ The development of total-body PET opens new radiotracer development opportunities in the context of acute and intensive care medicine, which in turn would allow us to gain a better understanding of how opportunistic infections initiate, progress, and respond to treatment.

Tangential to infection is the utility of immunoPET,^67^ which is likely to expand faster with total-body PET access because of the lesser concerns around injected mass of the radiotracer, as well as amenable imaging with less conventional radionuclides, such as zirconium-89. Furthermore, the potential to use immunoPET to better understand immune mapping in aging is unlocked by total-body PET low-dose regimens that would be more amenable to healthy human imaging. In this regard, recent projects proposing walk-through total-body radionuclide imaging may unlock the potential of PET in natural aging research and preventive medicine. In support of the use of total-body PET to unlock immune response understanding, studies are showing that this technology, coupled with selective PET radiotracers, can be used to understand adaptive immune response to COVID-19 viral infections and subsequent immunological memory for the development of vaccines.^68^

Another exciting and emerging field in radiotracer discovery that can be accelerated by total-body PET is nanobots^69^ and other stimulus-responsive release theranostic systems, which orchestrate controlled releases, enhancing precision and therapeutic effectiveness.^51^ Several safety considerations and strategies for mitigating side effects of these theranostic approaches have limited translation into humans, but cutting-edge innovations in next-generation radiotracers^51^ are breaking barriers to their clinical use. The use of total-body PET low-dose exploratory studies could not only provide important insights into the mechanisms of action of these new radiotracer species but also work as a platform for the assessment of target-off-target effects, allowing potential side effects of new theranostic radiotracers to be identified and then mitigated. Therefore, total-body PET technology could accelerate and promote new radiotracer discovery programs beyond classic small-molecule, peptide or antibody imaging. Furthermore, total-body PET technology could improve pharmacokinetics and pharmacodynamic analysis of nanodrugs in vivo behavior by enabling quantitatively accurate late-imaging studies (>24 hours). This is important because nanodrugs can have slow kinetics in vivo, and accurate quantification with conventional PET technology is challenging due to the limited sensitivity. Furthermore, nanodrugs’ safety profile may be more complex than other types of conventional small-molecule-based drugs (eg, kidneys radiotoxicity). Being able to administer these in lower doses, while still being able to track them in vivo in a quantitatively accurate manner, would allow for faster optimization of nanodrug delivery systems for subsequent widespread translation.

Cell tracking radiotracer development programs, albeit already possible with conventional PET scanners, could majorly benefit from the improved sensitivity of total-body PET technology,^1^ because a recurrent issue with radiotracers for cell tracking is the poor signal-to-noise ratio, with difficult subsequent interpretation of the results. Several total-body PET review papers exist, which explore additional applications of the new technology and provide good resources for further reflection on new targets for radiotracer discovery enabled by total-body PET.^1^^,^^70^, ^71^, ^72^

Whole-person research can be accelerated by total-body PET imaging with new or repurposed radiotracers. To date, radiotracer development efforts have been focused on single-organ imaging. This narrow view of whole-body functions limits a priori identification of patients most likely to beneficiate from new interventions, because it does not take into consideration multiple organ systems’ responses to a given treatment nor multitissue innate molecular/functional baselines in different patients. In this regard, total-body PET can support the development of new system-level methodologies for subsequent application in the clinical management of patients.

Although total-body PET imaging represents an important transformative step in nuclear medicine, conventional PET technology will retain its role in the clinical routine realm. This is not only because of past successes in the field of oncology, neurology, and cardiology, but also because the widespread presence of conventional PET technology is well established in the field. Likely, in the near future at least, total-body PET sites will play a pivotal role in research-focused, new methodologies development (including radiotracer development) that will benefit the entire PET field and see dissemination through the established conventional PET networks.

### G Three-tiered vision for total-body positron emission tomography in radiotracer development programs

The key impacts of total-body PET technology in radiotracer development programs can be classified into 3 main tiers: (1) “niche” impact, where sites with access to total-body PET scanners can lead the development of new radiotracers aiming to image targets with low expression that require high target-background ratios; (2) community impact, where sites with total-body PET technology facilitate development of PET radiotracers and associated quantification models that will then be rolled out to the many sites with standard PET; and (3) next-generation personalized medicine impact, where new radiotracers and models developed at total-body PET sites will act as companion biomarkers in personalized clinical interventions. For the community impact tier, total-body PET can help radiotracer development by measuring new radiotracer biokinetics at low dose and over long periods, in order to identify feasibility, specificity, best imaging protocols, and quantitative modeling strategies for subsequent dissemination to other sites with or without access to total-body PET technology. For the next-generation personalized medicine tier, total-body PET could aid drug and treatment development, thereby helping with overcoming another issue with the radiotracer “valley of death” that is the lack of impact of new radiotracers on clinical therapy guidance. It is known that once a therapy and radiotracer align on the same target, the community adoption of the PET biomarker to guide and personalize interventions is greater, consequently delivering on personalized medicine ethos.

## II Concluding remarks

Total-body PET technology is not only creating new opportunities for clinical research and patient care but also enabling radiochemistry modernization in the clinic and breaking down barriers for radiotracer discovery pipelines. It has tremendous potential to ameliorate regulatory hurdles and aid the delivery of the long-yearned era of personalized medicine, if the community can collaboratively redesign regulatory frameworks in conjunction with regulators. Although tangible practical testing of these theoretical advantages of total-body PET in radiotracer discovery pipelines has not yet happened, the potential is there and the future is bright if the community can come together to capitalize on the great potential of total-body PET in radiotracer discovery programs.

## Conflict of interest

Andrew Sutherland and Adriana A. S. Tavares disclose a patent (TSPO Binders, WO2019243616).
